# DEPRESSION ANXIETY STRESS SCALE IN OLDER ADULTS: ITEM RESPONSE THEORY AND CONFIRMATORY FACTOR MODELS

**DOI:** 10.1093/geroni/igae098.2601

**Published:** 2024-12-31

**Authors:** Wen Liu, M Bridget Zimmerman, Katherine Hadlandsmyth, Tracey Smith, Joseph A Buckwalter, David M Green, Lilian Dindo, Barbara Rakel

**Affiliations:** University of Iowa, Iowa City, Iowa, United States; University of Iowa, Iowa City, Iowa, United States; University of Iowa, Iowa City, Iowa, United States; Baylor College of Medicine, Houston, Texas, United States; University of Iowa, Iowa City, Iowa, United States; Baylor College of Medicine, Houston, Texas, United States; Baylor College of Medicine, Houston, Texas, United States; University of Iowa, Iowa City, Iowa, United States

## Abstract

The Depression Anxiety & Stress Scale (DASS) is a commonly used tool to assess patient-reported symptoms of depression, anxiety, and stress, and has been translated into multiple languages. This study examined the psychometric properties of DASS in older U.S. Veterans who experience knee pain prior to Total Knee Arthroplasty. A total of 660 Veterans (age 66.7 □ 9.1 years; 88% male; 72.2% white, 61% married) were consented and screened for enrollment in an on-going, double-blind, two-arm, randomized controlled trial, evaluating the efficacy of an Acceptance and Commitment Therapy intervention. All Veterans completed DASS during post-consent screening. Confirmatory Factor Analysis was conducted to examine the fit of the data to a 3-factor structure (Depression, Anxiety, Stress). After unidimensionality was confirmed, Item Response Theory (IRT) analysis was conducted to examine item difficulty and discrimination within each factor. The 3-factor structure showed desirable model fit such that there were strong correlations among three factors (r ranges =.837-.883, p<.001). The three factors showed acceptable to desirable internal consistency (Cronbach alpha=0.904 for Depression, 0.767 for Anxiety, 0.883 for Stress). DASS showed acceptable model fit as a 3-factor model to IRT. Items in each factor showed acceptable discriminations and spread evenly on difficulty levels. Item and test information curves were unimodal and nearly symmetric. DASS has been adopted worldwide as a self-report assessment of distress. Findings support the use of the 3-factor structure in older Veterans. Further research is needed to investigate performance of DASS in Veterans with more diversity in gender and socioeconomic status.

